# Investigation of cell culture conditions for optimal foot-and-mouth disease virus production

**DOI:** 10.1186/s12896-019-0527-5

**Published:** 2019-06-07

**Authors:** Veronika Dill, Aline Zimmer, Martin Beer, Michael Eschbaumer

**Affiliations:** 1 Institute of Diagnostic Virology, Friedrich-Loeffler-Institut, Südufer 10, 17493 Greifswald, Insel Riems Germany; 2 Merck KGaA, Merck Life Sciences, Upstream R&D, Frankfurter Straße, 250, 64293 Darmstadt, Germany

**Keywords:** Foot-and-mouth disease virus, Vaccine, Glucose, Glutamine, Cell density, Suspension cells, Animal-component free

## Abstract

**Background:**

Foot-and-mouth disease is a highly contagious and economically devastating disease with endemic occurrence in many parts of the world. Vaccination is the method of choice to eradicate the disease and to limit the viral spread. The vaccine production process is based on mammalian cell culture, in which the viral yield varies in dependence of the composition of the culture media. For foot-and-mouth disease virus (FMDV), very little is known about the culture media components that are necessary to grow the virus to high titers in cell culture.

**Results:**

This study examined the influence of increasing concentrations of glucose, glutamine, ammonium chloride and different cell densities on the yield of FMDV. While an excess of glucose or glutamine does not affect the viral yield, increasing cell density reduces the viral titer by a log_10_ step at a cell density of 3 × 10^6^ cells/mL. This can be mitigated by performing a 100% media exchange before infection of the cells.

**Conclusions:**

The reasons for the diminished viral growth, if no complete media exchange has been performed prior to infection, remain unclear and further studies are necessary to investigate the causes more deeply. For now, the results argue for a vaccine production process with 100% media exchange to reliably obtain high viral titers.

**Electronic supplementary material:**

The online version of this article (10.1186/s12896-019-0527-5) contains supplementary material, which is available to authorized users.

## Background

Foot-and-mouth disease (FMD) is a viral disease of cloven-hoofed livestock with tremendous economic impact [1]. Every year, more than one billion doses of FMD vaccine are produced worldwide [2]. These vaccines are used for control programs in regions where FMD is endemic and for the emergency response to outbreaks in areas where the disease does not occur regularly [2, 3].

Mammalian cells are widely used for the propagation of viruses for vaccine production. The viral yield in cell culture varies greatly depending on the composition of the culture media [4]. For instance, the production of poliovirus in HeLa cells differs with the media composition, with salts, glucose and glutamine representing the only essential substrates for successful virus production [4, 5]. Glucose and glutamine are the main carbon sources for mammalian cells in culture. They are key nutrients to cover the cell’s energy requirements [6]. The glycolysis and glutaminolysis pathways in the cell are utilized at high rates to metabolize these substrates, leading to the production of high amounts of waste products such as lactate and ammonium [7]. Many viral infections are characterized by an increase in the rate of glycolysis, e.g. poliomyelitis virus [8], feline leukemia virus [9], or herpes simplex virus [10] or an increase in glutamine uptake, e.g. vaccinia virus [11] or human cytomegalovirus [12]. In addition to these two important pathways, a viral infection of the cell leads to other changes in the cellular metabolism, such as fatty acid synthesis [13, 14].

For foot-and-mouth disease virus (FMDV), very little is known about the culture media components that are necessary to grow the virus to high titers in cell culture. An early study by Pledger et al. [15] named glucose as an important substrate for FMDV replication, while glutamine alone had no influence on the viral titer. However, the metabolism of glucose produces high amounts of lactate that are released into the culture media, decreasing its pH if the buffer capacity of the media is exceeded [13, 16]. Optimizing the glucose and glutamine content of the culture media can increase the viral harvest, while the use of increased cell densities allows a more efficient use of bioreactor capacity and decreased costs per dose of vaccine [16]. Using animal-component free (ACF) media for vaccine production can further reduce costs and minimize the risk of contamination through animal-derived raw materials [3, 17].

This study investigated the metabolism of baby hamster kidney (BHK) suspension cells infected with the recent FMDV isolates A IRN/8/2015 and O SAU/18/2015. The viral titer as well as the effect of different concentrations of glucose, glutamine and ammonium chloride in the medium were examined. Furthermore, infection at different cell densities in combination with a total or partial media exchange was compared.

## Results

### Abundant glucose and glutamine in the cell culture media does not increase the viral yield

**Fig. 1:**
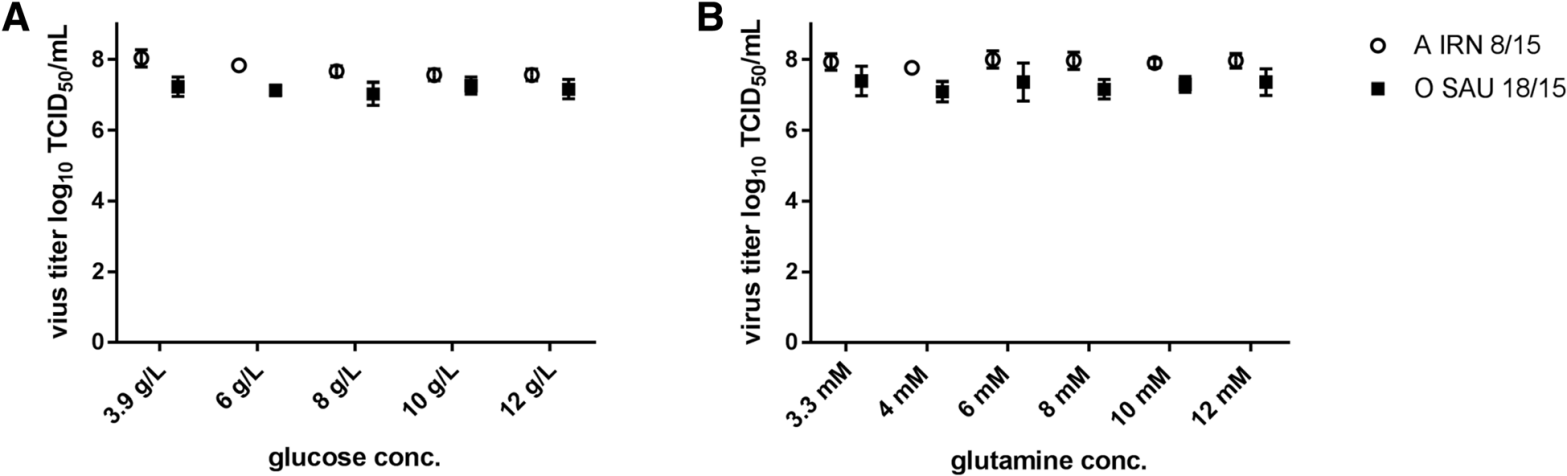
Viral yield of BHK-2P cells maintained in ACFM with increasing concentrations of glucose (**a**) or glutamine (**b**). Cells were infected with FMDV A IRN/8/2015 (open circles) or O SAU/18/2015 (filled squares). No differences in viral titer were observed for any of the tested conditions. Experiments were performed three times independently and were titrated in duplicates

Infection studies of BHK-2P suspension cells were performed using the recent FMDV isolates A IRN/8/2015 and O SAU/18/2015 and increasing concentrations of glucose and glutamine in the cell culture media. Independently of the glucose or glutamine concentration, stable virus titers of 7.7 ± 0.2 log_10_ TCID_50_/mL across the different glucose concentrations and 7.9 ± 0.1 log_10_ TCID_50_/mL across the different concentrations of glutamine were achieved for A IRN/08/2015, compared to 7.2 ± 0.1 log_10_ TCID_50_/mL and 7.3 ± 0.1 log_10_ TCID_50_/mL for O SAU/18/2015, respectively (Fig. 1).

**Fig. 2:**
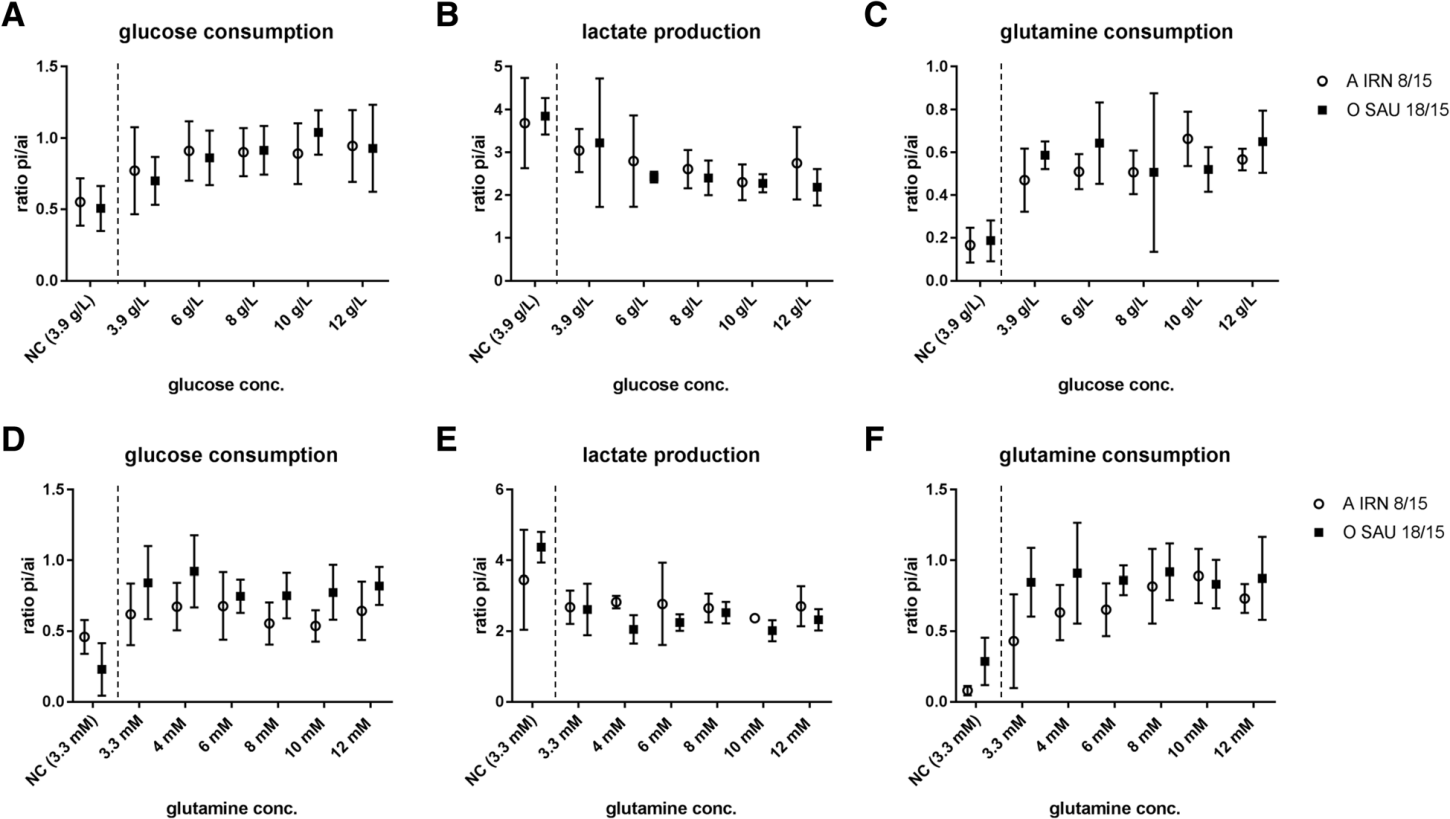
Difference in content of glucose,glutamine and lactate in with increasing glucose and glutamine concentration in media. BHK-2P cells were maintained in ACF media with increasing concentrations of glucose (**a**-**c**) or glutamine (**d**-**f**) and infected with either A IRN/8/2015 (open circles) or O SAU/18/2015 (filled squares). Experiments were performed three times independently. The glucose content was determined in duplicate for every experiment. The lactate and glutamine contents were determined three times individually. No statistically significant differences in the difference of content of glucose or glutamine or lactate were detected between the different conditions. The y-axis presents the ratio between the concentration of each metabolite at 20 hpi (pi) and at the beginning of the experiment (ai)

No statistically significant differences in the content of glutamine and glucose in the cell culture media before and after viral infection were observed in any of the tested conditions for either virus isolate (Fig. 2).

Cell viability in the infected cultures dropped drastically after 20 hpi compared to the uninfected negative controls in all experiments (see Additional file 1). The amounts of lactate accumulated in the media of the infected cultures were 10 to 60% lower (depending on serotype and tested condition) in comparison to the negative control. Nevertheless, among the infected cultures, no statistically significant difference between the tested conditions was detected (Fig. 2).

In general, the cells consumed only a small fraction of the available carbohydrates when infected with FMDV based on the differences in the glucose and glutamine content of the media before and after viral infection independent of the total concentration of glucose or glutamine in the media. The difference in concentration of glutamine and glucose for the respective negative controls was nearly twice as much than for the infected cells (Table 1).

**Table 1:** Mean glucose and glutamine content in the cell culture media of BHK-2P suspension cells when infected with FMDV

cells infected with	A IRN 8/15	O SAU 18/15	NC
glucose (g/L)	1.7 ± 0.5	1.0 ± 0.6	2.7 ± 1.1
glutamine (mM)	1.8 ± 0.7	1.5 ± 0.6	2.9 ± 0.4

Cells were either infected with FMDV A IRN/8/2015 or O SAU/18/2015 or uninfected (NC = negative control). The content was calculated by subtracting the measured concentration of the analyte at 20 hpi from the measured concentration before infection

Calculations of the cell-specific glucose uptake rates revealed a positive trend of an increased glucose consumption with increased glucose availability when infected with FMDV serotype A that was not evident for cells infected with serotype O (see Additional file 3: Table S3). On the other hand, the data for the cell-specific glutamine uptake suggest an increased uptake of glutamine with increasing concentrations of glutamine available in the cell culture media when infected with serotype O, while no trend was evident for serotype-A-infected cells (see Additional file 3: Table S4).

#### The concentration of ammonium chloride in the cell culture media influences cell survival but not the viral yield

**Fig. 3:**
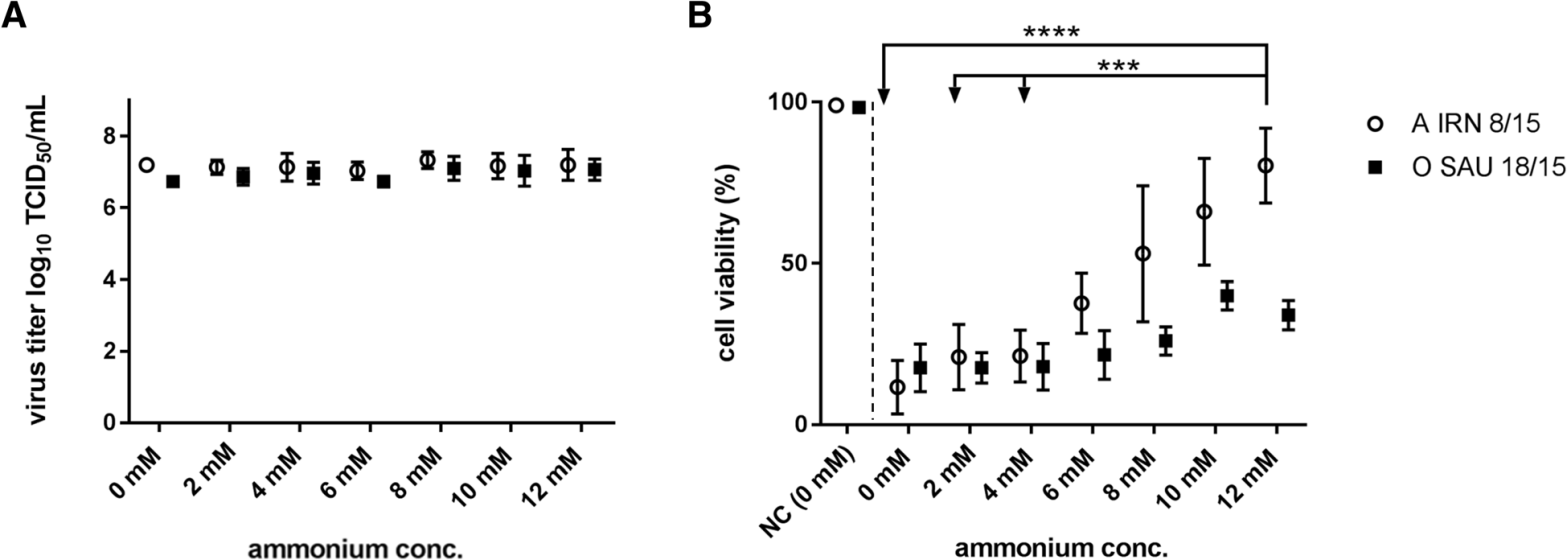
Viral titers of BHK-2P cells maintained in ACF media with increasing concentrations of ammonium chloride. Cells were infected with FMDV A IRN/8/2015 (open circles) or O SAU/18/2015 (filled squares). No difference in viral titer (**a**) was observed for any of the tested conditions. Cell viability 20 hpi (**b**) increased with increasing concentration of ammonium chloride in the cell media. Experiments were performed three times independently and the viral yield was titrated in duplicate. Significance code: *** *p* < 0.001; **** *p* < 0.0001

The ACF media was supplemented with increasing concentrations of ammonium chloride ranging from 0 mM to 12 mM and cells were infected with A IRN/8/2015 or O SAU/18/2015. Surprisingly, the viral yield was stable at a titer of 7.2 ± 0.1 log_10_ TCID_50_/mL for A IRN 8/2015 and 6.9 ± 0.2 log_10_ TCID_50_/mL for O SAU 18/2015, while cell viability at 20 hpi increased with increasing concentrations of ammonium chloride. That increase was statistically significant in the case of A IRN 8/15 (Fig. 3).

**Fig. 4:**
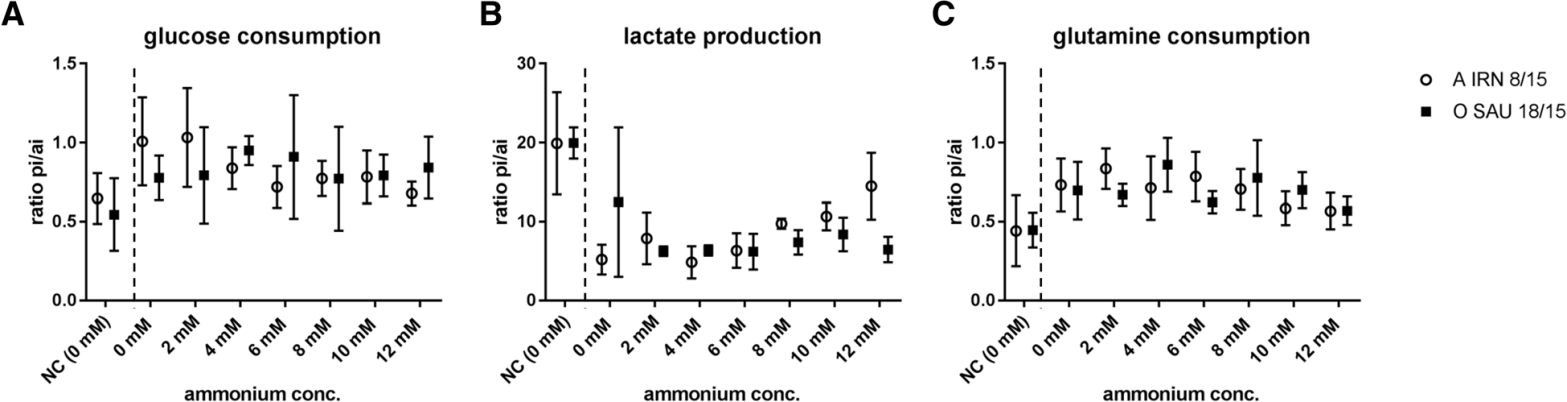
Difference in content of glucose (**a**), lactate (**b**) and glutamine (**c**) in the cell culture media with increasing concentrations of ammonium chloride. BHK-2P cells were maintained in ACF media and infected with either A IRN/8/2015 (open circles) or O SAU/18/2015 (filled squares). Experiments were performed three times independently. The content of glucose was determined in duplicates for every experiment. The content of lactate and glutamine was determined three times individually. No statistically significant differences in the difference of content for glucose,glutamine or lactate could be detected between the conditions in infected cells. Nevertheless, an increase in the contents of glutamine and lactate is evident at ammonium chloride concentrations above 6 mM

The prolonged survival of the infected BHK-2P cells at or above an ammonium chloride concentration of 6 mM is reflected in an increased content of glutamine and glucose as well as lactate in the cell culture media (Fig. 4). The difference in the content of glucose in the cell culture media before and after infection does not vary strongly between infected and uninfected cells but the content of lactate is reduced in the cell culture media of FMDV-infected cells. The calculation of the cell-specific glucose and glutamine uptake as well as lactate production revealed an increasing trend for all three metabolites between a concentration of 2–10 mM NH_4_Cl in the media for cells infected with the FMDV A/IRN/8/2015 isolate, while no such trend was evident for cells infected with FMDV O/SAU/18/2015 (see Additional file 3: Table S5).

#### Increased cell density leads to decreased viral yield

**Fig. 5:**
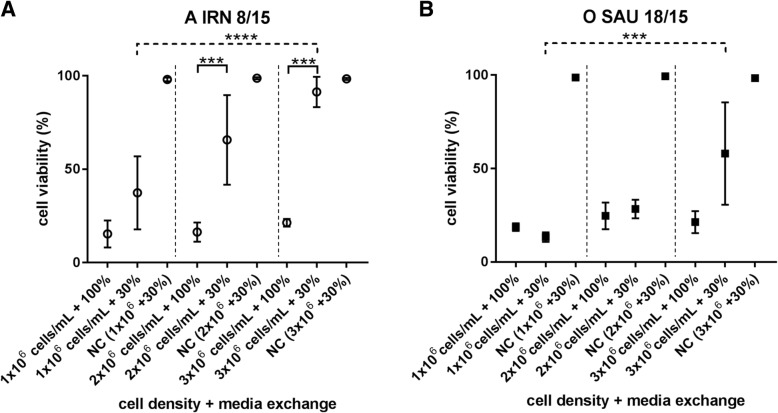
Cell viability of BHK-2P cells at increasing densities and different media substitutions. A substitution of 30% or 100% fresh media was performed before infection. Cells were infected with FMDV A IRN/8/2015 (**a**) or O SAU/18/2015 (**b**). Significant differences in cell viability 20 hpi were detected between 30 and 100% media substitution at cell densities of 2 × 106 cells/mL and 3 × 106 cells/mL for A IRN/8/2015 and between the 30% media exchange preparations of 1 × 106 cells/mL and 3 × 106 cells/mL for O SAU/18/2015. Experiments were performed three times independently. Significance code: *** *p* < 0.001; **** *p* < 0.0001

**Fig. 6:**
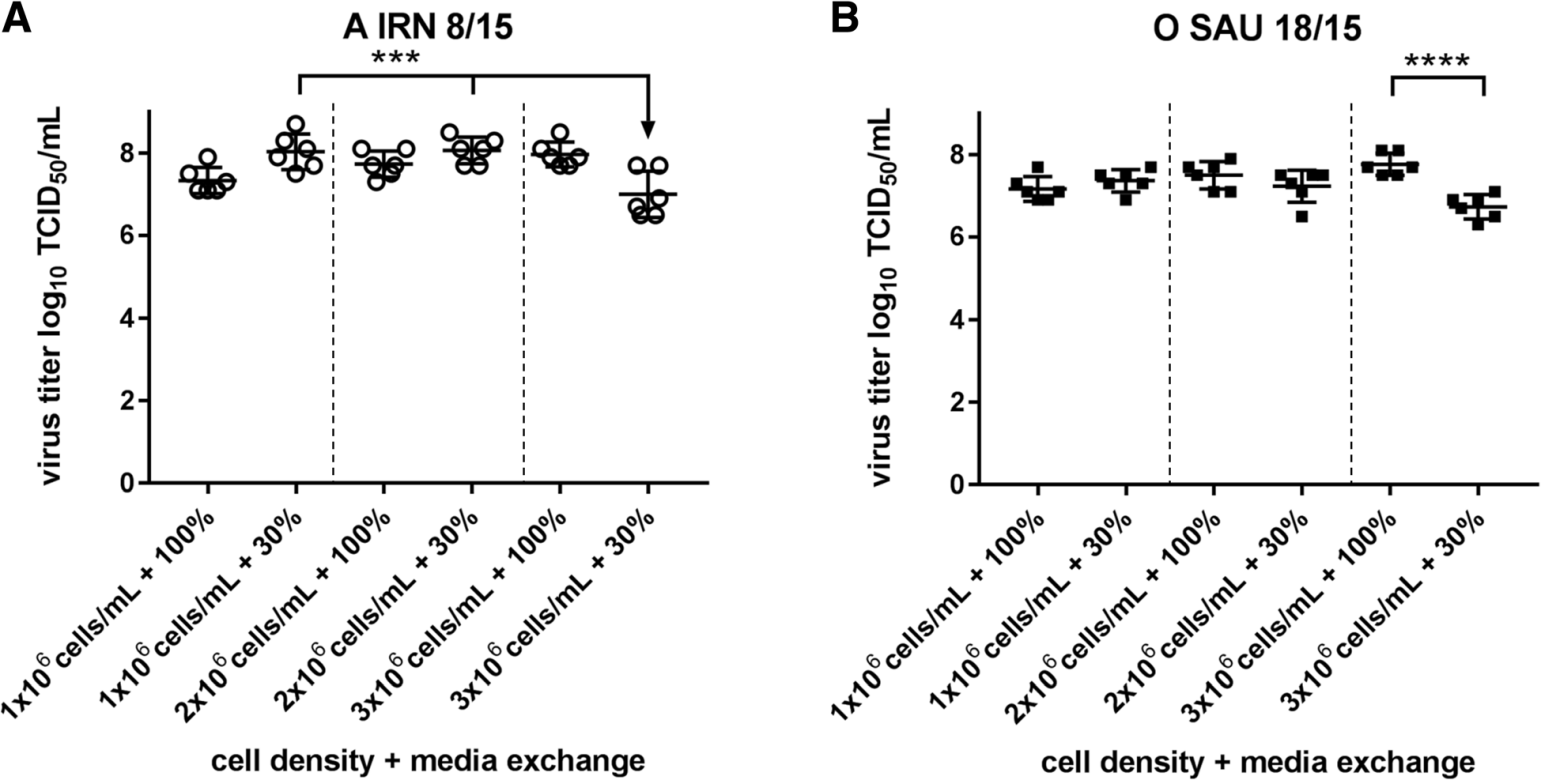
Viral yield of BHK-2P cells at increasing densities and different media substitutions. BHK-2P cells were maintained in ACF media with increasing cell densities and a 30% or 100% media exchange before infection. Then cells were infected with FMDV A IRN/8/2015 (**a**) or O SAU/18/2015 (**b**). Viral titers for both isolates were significantly reduced at a cell density of 3 × 106 cells/mL if only 30% of media was exchanged. Experiments were performed three times independently and the viral yield was titrated in duplicate. Significance code: *** *p* < 0.001; **** *p* < 0.0001

Cell densities of 1 × 10^6^ cells/mL, 2 × 10^6^ cells/mL and 3 × 10^6^ cells/mL were infected with FMDV A IRN/8/2015 or O SAU/18/2015, either with a media exchange of 100% or with a media exchange of 30% before infection. Cell viability at 20 hpi was higher (Fig. 5) and viral titers were lower (Fig. 6) at higher cell densities when only 30% of the culture media was replaced with fresh media before infection. While differences in cell viability 20 hpi between 100 and 30% media exchange are already evident for A IRN/8/2015 at a cell density of 2 × 10^6^ cells/mL, a statistically significant difference in cell death was only seen between the 30% media exchange preparations of 1 × 10^6^ cells/mL and 3 × 10^6^ cells/mL for O SAU/18/2015 (Fig. 5). No differences in the cell viability were detected when the full volume of medium was exchanged, independently of the cell density.

Viral titers of 7.7 ± 0.3 log_10_ TCID_50_/mL for A IRN/8/2015 and 7.5 ± 0.3 log_10_ TCID_50_/mL for O SAU/18/2015 were achieved at all tested cell densities if a 100% media exchange was performed. A slight increase in viral titer with increasing cell density was seen, but it was not statistically significant (see Additional file 2: Table S1). With a 30% media exchange before infection, stable titers of 8.1 ± 0.0 log_10_ TCID_50_/mL for A IRN/8/2015 and 7.3 ± 0.1 log_10_ TCID_50_/mL for O SAU/18/2015 were reached for densities of 1 × 10^6^ cells/mL and 2 × 10^6^ cells/mL, while the viral titer significantly dropped by one log_10_ at a cell density of 3 × 10^6^ cells/mL (serotype A: 7.0 ± 0.6 log_10_ TCID_50_/mL; serotype O: 6.7 ± 0.3 log_10_ TCID_50_/mL) (Fig. 6).

The difference in the content of glucose, lactate and glutamine before and after viral infection was similar across the different cell densities when 100% of media were exchanged. Similar to the experiments with increasing glucose and glutamine concentrations in the media, only a small fraction of the provided carbohydrates was metabolized by the virus-infected cells. With an incomplete exchange of media (30%), the difference in the contents of glucose and lactate in the cell culture media of the virus-infected cell cultures were similar to the negative controls. High values of lactate (> 10 mM) and a decreased pH of the culture media were already evident at a cell density of 1 × 10^6^ cells/mL (Table 2). The depletion of glutamine in the virus-infected cell cultures with 30% media exchange was slightly less compared to the negative controls of the same cell density (Table 2). In general, glutamine in the negative cultures was almost completely depleted. The comparison of the cell-specific uptake rates showed a negative trend for the mock-infected cells and cells infected in 30% fresh media with reduced glucose consumption at higher cell densities, whereas cells infected in 100% fresh media increased their glucose uptake (see Additional file 3: Table S6).

**Table 2:** Glucose, lactate, and glutamine content and pH of the ACF media 20 h after viral infection

virus	A IRN/8/2015		NC	A IRN/8/2015		NC	A IRN/8/2015		NC
cell density	1 × 10^6^ cells/mL			2 × 10^6^ cells/mL			3 × 10^6^ cells/mL		
media exchange	100%	30%	30%	100%	30%	30%	100%	30%	30%
pH	7.5 ± 0.1	7.3 ± 0.1	7.1 ± 0.2	7.3 ± 0.1	7.0 ± 0.1	7.0 ± 0.1	7.2 ± 0.1	7.0 ± 0.3	6.9 ± 0.1
glucose (g/L)	5.2 ± 1.2	3.4 ± 1.0	2.6 ± 0.3	3.7 ± 1.8	1.6 ± 0.4	1.4 ± 0.6	2.6 ± 1.0	1.1 ± 0.5	1.2 ± 0.6
lactate (mM)	8.2 ± 0.9	23.0 ± 6.1	26.9 ± 4.6	16.6 ± 3.1	28.5 ± 4.1	29.2 ± 3.5	18.5 ± 2.6	25.1 ± 5.3	33.6 ± 4.1
glutamine (mM)	2.5 ± 1.8	1.5 ± 1.0	0.4 ± 0.3	2.5 ± 0.9	0.6 ± 0.6	0.1 ± 0.1	1.6 ± 0.6	0.3 ± 0.3	0.7 ± 0.9
virus isolate	O SAU/18/2015		NC	O SAU/18/2015		NC	O SAU/18/2015		NC
cell density	1 × 10^6^ cells/mL			2 × 10^6^ cells/mL			3 × 10^6^ cells/mL		
media exchange	100%	30%	30%	100%	30%	30%	100%	30%	30%
pH	7.4 ± 0.0	7.4 ± 0.0	7.1 ± 0.0	7.4 ± 0.0	7.2 ± 0.2	7.0 ± 0.2	7.2 ± 0.20	6.9 ± 0.2	6.9 ± 0.2
glucose (g/L)	7.0 ± 1.6	5.2 ± 1.0	3.5 ± 1.0	5.1 ± 0.7	3.1 ± 1.2	2.1 ± 0.8	4.1 ± 0.3	1.5 ± 0.9	1.6 ± 0.5
lactate (mM)	7.5 ± 4.1	11.5 ± 5.8	21.4 ± 10.7	14.9 ± 6.8	23.7 ± 11.1	27.9 ± 14.0	16.6 ± 8.4	29.2 ± 14.9	36.8 ± 19.1
glutamine (mM)	4.3 ± 1.5	3.1 ± 0.9	1.3 ± 0.3	4.0 ± 1.3	1.6 ± 0.7	0.5 ± 0.4	2.6 ± 0.5	0.7 ± 0.3	0.2 ± 0.1

## Discussion

Several studies have reported an increased uptake of glutamine and/or glucose in the course of a viral infection of cell cultures [13, 14]. Very little is known about the metabolic processes in an FMDV-infected cell culture or about the nutrient requirements for successful virus production. For poliomyelitis virus, another picornavirus, it is known that virus production depends on the media composition [4]. For that virus, it was hypothesized that glucose and glutamine are necessary as energy sources or for synthesis of the viral nucleic acid [5]. Of the two nutrients, glutamine was determined to be the more important factor, possibly due to the high inherent glutaminase activity of HeLa cells [4].

Conversely, for FMDV, an early study by Pledger and colleagues determined glucose as the only necessary factor for virus replication in primary bovine kidney cells [15]. The present study examined the possibility of increasing the FMD viral yield by providing more of these key nutrients in the culture media, but neither an increased concentration of glucose (up to 12 g/L) nor of glutamine (up to 12 mM) had a significant effect on the virus titer. In addition, no increase in glycolysis or glutaminolysis of the cell was observed. Darnell and colleagues have proposed that the optimal glucose and glutamine concentrations for virus production are the same as for cell growth [4]. Comparing the residual content of glucose and glutamine in the media between a mature infected culture and the corresponding negative control revealed that only a small fraction of the supplied nutrients had been metabolized. As previously proposed by Pledger et al., extracellular nutrient requirements seem to be of lesser importance for FMD virus particle production [15]. A possible explanation for this is the rapid growth of FMDV in infected cells. While one replication cycle of poliovirus takes 3–7.5 h (depending on the cell line), FMDV only needs 1.5 to 2.5 h to replicate in primary bovine kidney cells [15] and the first newly produced virus particles usually appear in the culture media after 4–6 h [18]. Due to the rapid progression of lytic infection, most cells in the culture vessel are dead before the available nutrients are exhausted. Consequently, metabolic waste products such as lactate were not sufficiently increased to lower the pH to a level that would negatively impact the viral yield. FMDV particles are highly acid-sensitive and the capsid dissociates at pH values slightly below neutrality (< pH 6.8) [19]. In the course of infection in this study, the pH of the media decreased from 7.5 to at most 7.2, which has no impact on the stability of virus particles.

To initiate infection, FMDV releases its genome into the host cell by dissociation of the capsid into pentameric subunits, which is triggered by the acidification of the endosome [20]. Ammonium ions (NH4^+^), a waste product of the glutamine metabolism pathway, neutralize the acidic pH within endosomes and block the uncoating of the viral RNA [21]. Surprisingly, we found no difference in the viral titers in cultures supplemented with increasing concentrations of ammonium chloride (up to 12 mM). At the same time, the cell viability in infected cultures increased dramatically at higher concentrations of ammonium chloride, particularly for the serotype A virus isolate.

Several studies have been performed to examine the effect of lysosomotropic agents on FMDV infection [20–23]. In most cases, only concentrations of 25 mM and 50 mM ammonium chloride were tested and these resulted in a drastically reduced viral titer at 25 mM and a complete block of infection at 50 mM NH_4_Cl [20]. Carillo et al. tested the influence of lower concentrations of ammonium chloride (10, 20 and 30 mM), leading to a 95% reduction in the viral yield for 30 mM and a proportionally lower impact for 20 mM and 10 mM [21]. No data are available for batch cultures of BHK cells infected with FMDV, but glutamine metabolism and ammonium production during cell growth and virus infection in batch culture are well studied for MDCK cells and influenza virus [24]. Similar to the observations for conventional FMDV culture, a reduction in the yield of influenza virus was evident for NH_4_Cl concentrations above 20 mM. However, this is not likely to have an effect under real production conditions. In theory, a maximum of 4 mM ammonium can be produced from the 4 mM glutamine available at the start of the process [24], but in the experiment, no more than 2 mM of ammonium were produced by the cells throughout the entire process [24]. Considering the rapid progression of FMDV infection, it is unlikely that the real ammonium concentrations produced during FMDV antigen production exceed these values and thus influence the yield. Nevertheless, in the absence of a satisfactory explanation for the increased cell viability observed at higher concentrations, production processes should be controlled in such a way that not more than 6 mM ammonium can accumulate in the culture media before viral infection.

Several studies have reported so-called “cell density effects” in different culture systems used for virus production [16, 25, 26]. For our studies, three different cell densities (1 × 10^6^ cells/mL, 2 × 10^6^ cells/mL and 3 × 10^6^ cells/mL) with either 100% or 30% media exchange before infection were tested. No difference in viral titer was detected between the different cell densities if a full media exchange was performed. An exchange of 30% of the media led to a high variability in cell viability at the end of the process and together with a cell density of 3 × 10^6^ cells/mL reduced the final viral titer by about one log_10_ step. This may be due to nutrient limitation or the accumulation of inhibitory factors [16, 25], but the exact cause of the specific “cell density effect” observed in our system is unknown. The lactate content was elevated in cultures with 3 × 10^6^ cells/mL and 30% media exchange, but it is unlikely that this directly influenced infectivity. The pH of the culture medium was adjusted to pH 7.5 before infection and high concentrations of lactate alone do not impair the growth of BHK cells [27]. With only 30% of media being replaced, glucose and especially glutamine levels are reduced compared to cultures with a full exchange, but the available nutrients should be still sufficient for virus production. Further experiments are needed to examine whether this effect is still visible if the concentrations of glucose and glutamine are increased at the time of infection.

The examination of cell growth rates at the different conditions could also offer valuable information about the ability of the cells to produce virus. An inhibited cell growth can be linked to reduced virus production. In addition, it is known that the impact of excessive cell densities on production depends on the culture medium [25]. It would be of interest if other culture media can mitigate the “cell density effect” and further improve FMDV vaccine production processes.

## Conclusions

This study determined important culture parameters that influence FMDV titers in a small-scale bioreactor system. While an excess of glucose, glutamine and ammonia in the culture media does not directly influence the viral yield, the cell density seems to have the largest impact on the viral titers achieved in batch culture. Further experiments have to be performed to study the nature of this effect in greater detail. Based on the current state of knowledge, 100% media exchange is recommended for optimal yield and high reproducibility.

## Methods

All experiments have been performed in a veterinary BSL-4 laboratory that meets the Minimum Biorisk Management Standards for Laboratories Working with FMDV [28].

### Cells

The suspension cell line BHK21C13-2P (in short: BHK-2P; originally derived from the European Collection of Authenticated Cell Cultures specimen 84,111,301) was adapted to grow in Cellvento™ BHK-200 (Merck KGaA, Darmstadt, Germany) ACF medium in TubeSpin® bioreactors (TPP Techno Plastic Products AG, Trasadingen, Switzerland). The cells were maintained in a shaker incubator at 320 rpm (rpm) at 37 °C, 5% CO_2_ and 80% relative humidity.

For virus titrations, the adherent BHK21 clone “Tübingen” cell line (in short: BHK164, specimen CCLV-RIE 164 in the Collection of Cell Lines in Veterinary Medicine, Friedrich-Loeffler-Institut [FLI], Greifswald, Germany) was cultured in Minimum Essential Medium Eagle, supplemented with Hanks’ and Earle’s salts (Sigma-Aldrich, St. Louis, USA) with 5% fetal bovine serum. The adherent cells were incubated at 37 °C and 5% CO_2_.

### Viruses and virus titrations

The FMDV isolates A IRN/8/2015 and O SAU/18/2015 were selected from archival stocks at the FLI. Their origin and passage history can be found in Additional file 2: Table S2 Viral titers were estimated by endpoint titration with the Spearman-Kärber method [29, 30] and expressed as 50% tissue culture infectious dose (TCID50) per milliliter. Virus titrations were performed on the adherent BHK164.

### Small-scale bioreactor experiments

All experiments were performed in TubeSpin Bioreactors-50 (TPP) with a working volume of 30 ml. pH measurements before infection were performed with a potentiometric pH meter (Mettler Toledo, Gießen, Germany) and if necessary the pH was adjusted to 7.5 using 1 M sodium hydroxide. Cells were infected at a multiplicity of infection (MOI) of 0.1, and virus was harvested after 20 h of incubation. Virus samples were stored at − 70 °C, then thawed for further processing and cell debris were removed by centrifugation at 3200×g for 10 min at 4 °C. Samples to determine the content of glucose, lactate and glutamine were taken from the bioreactor immediately before infection and again 20 h post infection (hpi). These samples were centrifuged at 155×g for 5 min at 4 °C and the supernatant was stored at − 70 °C until further processing. pH measurements 20 hpi were performed using single-use pH test strips (range 6.5–10, Merck KGaA, Darmstadt, Germany) for biosafety reasons.

#### Glucose

TubeSpin cultures were seeded with 0.5 × 10^6^ cells/mL. The cells were pelleted at 290×g for 5 min and resuspended in 100% fresh media with different glucose concentrations (3.9 g/L, 6 g/L, 8 g/L, 10 g/L, 12 g/L). The cultures were placed in the shaker incubator overnight until a cell density of 1 × 10^6^ cells/mL was reached. Then the cells were pelleted again and 30% of the culture media were replaced with fresh media supplemented with the respective glucose concentration. After the 30% media exchange, the pH was adjusted to 7.5 if necessary and the cells were infected with FMDV A IRN/8/2015 or O SAU/18/2015. An uninfected negative control with standard conditions (3.9 g/L glucose) was also included. The experiment was performed three times independently.

#### Glutamine

TubeSpin cultures were seeded with 0.5 × 10^6^ cells/mL, cells were pelleted at 290×g for 5 min and resuspended in 100% fresh media with different glutamine concentrations (3.3 mM, 4 mM, 6 mM, 8 mM, 10 mM, 12 mM). The cultures were placed in the shaker incubator overnight until a cell density of 1 × 10^6^ cells/mL was reached. Then the cells were pelleted again and 30% of the culture media were replaced with fresh media, supplemented with the respective glutamine concentration. After the 30% media exchange, the pH was adjusted to 7.5 if necessary and the cells were infected with FMDV A IRN/8/2015 or O SAU/18/2015. An uninfected negative control with standard conditions (3.3 mM glutamine) was also included. The experiment was performed three times independently.

#### Ammonium chloride

TubeSpin cultures were inoculated with 1 × 10^6^ cells/mL, the cells were pelleted at 290×g for 5 min and resuspended in 100% fresh media supplemented with different concentrations of ammonium chloride (0 mM, 2 mM, 4 mM, 6 mM, 8 mM, 10 mM, 12 mM). After resuspension of the cells, the pH was adjusted to 7.5 if necessary and the cells were infected with FMDV A IRN/8/2015 or O SAU/18/2015. An uninfected negative control with standard conditions (no ammonium chloride) was also included. The experiment was performed three times independently.

#### Cell density

TubeSpin cultures were inoculated with 0.5 × 10^6^ cells/mL, 1.0 × 10^6^ cells/mL or 1.5 × 10^6^ cells/mL. The cells were pelleted at 290×g for 5 min and resuspended in 100% fresh media. The cultures were placed in the shaker incubator overnight to reach cell densities of 1 × 10^6^, 2 × 10^6^ and 3 × 10^6^ cells/mL, respectively. Then the cells were pelleted again and a media exchange of 100% or 30% was performed. After resuspension of the cells, the pH was adjusted to 7.5 if necessary and the cells were infected with FMDV A IRN/8/2015 or O SAU/18/2015. An uninfected negative control was also included. In total, three tubes per density were prepared: one tube with virus and 30% media exchange, one tube with virus and 100% media exchange, and one tube with no virus and 30% media exchange. The experiment was performed three times independently.

### Assays

Cell concentration and viability were assessed using a trypan blue dye exclusion method with an automated cell counter (TC20™, Bio-Rad, Munich, Germany). Glucose, lactate and glutamine concentrations were determined using quantitative colorimetric assays (EnzyChrom™ EBGL-100, EGLN-100 or ECLC-100, BioAssay Systems, Hayward, USA) as directed by the manufacturer.

### Statistical analysis

In all experiments, the differences between treatment groups were evaluated using one-way ANOVA, combined with Tukey’s multiple comparisons test in GraphPad Prism 7 (www.graphpad.com). *p*-values of < 0.001 were considered significant.

## Additional files

Additional file 1: **Figure S1**. Cell viability 20 hpi with FMDV in media with increasing concentrations of glucose (A) or glutamine (B). (TIF 770 kb)

Additional file 2: **Table S1.** Mean viral titers and standard deviation of cell density experiments. **Table S2.** Overview about the original virus isolates used in this study, their origin and passage history. (XLS 44 kb)

Additional file 3: **Table S3.** Cell-specific uptake and release rates of extracellular metabolites of infected BHK-2P cells and corresponding negative control with increasing extracellular glucose concentrations. **Table S4.** Cell-specific uptake and release rates of extracellular metabolites of infected BHK-2P cells and corresponding negative control with increasing extracellular glutamine concentrations. **Table S5.** Cell-specific uptake and release rates of extracellular metabolites of infected BHK-2P cells and corresponding negative control with increasing extracellular ammonium concentrations. **Table S6.** Cell-specific uptake and release rates of extracellular metabolites of infected BHK-2P cells and corresponding negative control at different cell densities and media exchange strategies. (XLSX 18 kb)

## Data Availability

All data generated or analysed during this study are included in this published article. Raw data are available from the corresponding author on reasonable request.

## Abbreviations

ACF: animal-component free
ANOVA: analysis of variance
BHK: baby hamster kidney; BHK-2P: BHK21C13-2P
CCLV-RIE: Collection of Cell Lines in Veterinary Medicine
FLI: Friedrich-Loeffler-Institut
FMD(V): foot-and-mouth disease (virus)
hpi: hours post infection
MDCK: Madin-Darby Canine Kidney
MOI: multiplicity of infection
NH_4_Cl: ammonium chloride
RNA: ribonucleic acid
TCID_50_: 50% tissue culture infectious dose
