# Supplementary figures and images for: Investigation of cell culture conditions for optimal foot-and-mouth disease virus production
Source: BMC Biotechnol. 2019 Jun 7;19:33. doi: 10.1186/s12896-019-0527-5 (PMC6555971; doi:10.1186/s12896-019-0527-5)

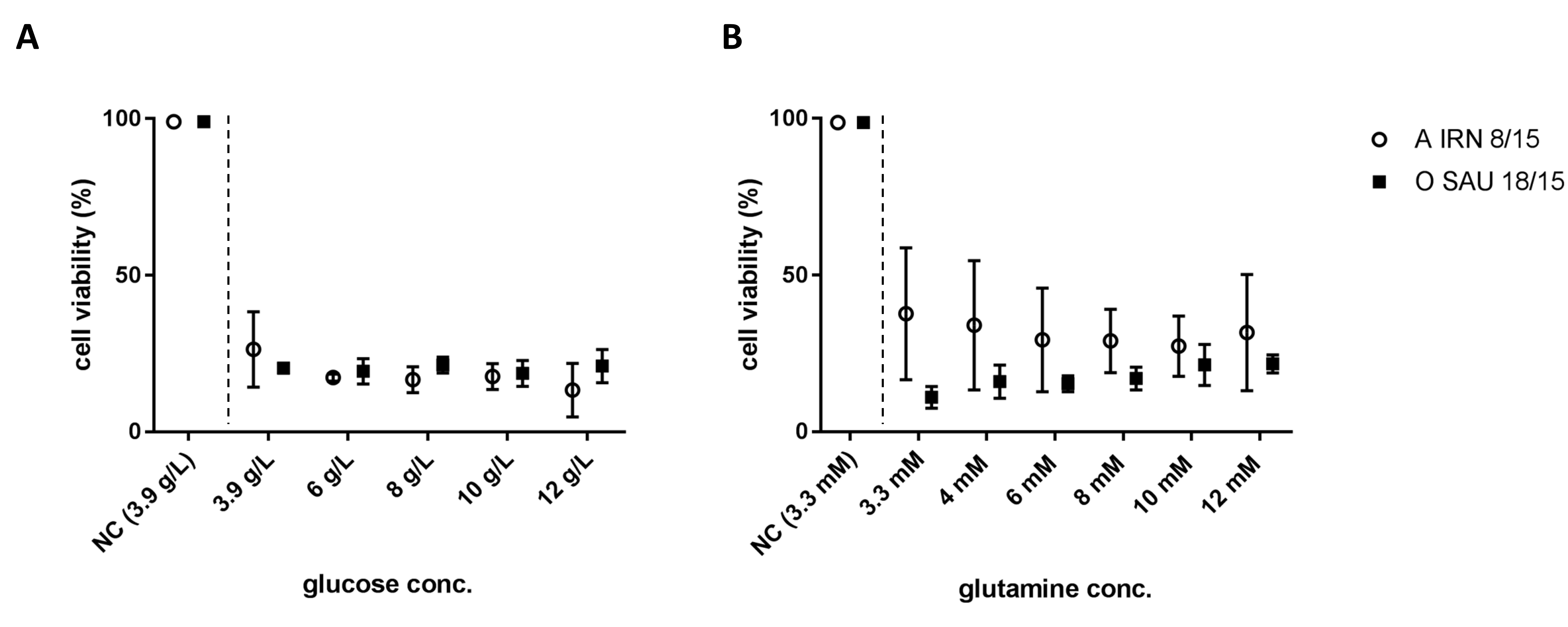

Supplement: Supplementary file 1 — Figure S1. Cell viability 20 hpi with FMDV in media with increasing concentrations of glucose (A) or glutamine (B). (TIF 770 kb) [file 12896_2019_527_MOESM1_ESM.tif]
